# Early Diabetic Retinopathy Evaluation With OCTA: A Study on Vascular Branching and Fragmentation

**DOI:** 10.1167/iovs.65.14.21

**Published:** 2024-12-10

**Authors:** Yao Yu, Shiwei Cui, Yang He, Jiahao Zhang, Nan Lu, Yanqiu Yang, Jian Liu, Yi Wang, Zhenhe Ma

**Affiliations:** 1 School of Control Engineering, Northeastern University at Qinhuangdao, Qinhuangdao, China; 2 Hebei Key Laboratory of Micro-Nano Precision Optical Sensing and Measurement Technology, Qinhuangdao, China; 3 Department of Ophthalmology, The First Hospital of Qinhuangdao, Qinhuangdao City, Hebei Province, China

**Keywords:** diabetic retinopathy (DR), optical coherence tomography angiography (OCTA), branching pattern, vascular fragmentation

## Abstract

**Purpose:**

The purpose of this study was to quantitatively evaluate the branching patterns and vascular fragmentation features in preclinical and early diabetic retinopathy (DR) using optical coherence tomography angiography (OCTA).

**Methods:**

OCTA metrics, including branch node number (BNN), branch node density (BND), end point number (EPN), end point density (EPD), fragmented vessel segment count (FVSC), and fragmented vascular length ratio (FVLR), were measured in foveal and parafoveal regions within superficial and deep vascular plexus (SVP and DVP) in the retina.

**Results:**

Compared to healthy control (HCs), both BNN and BND exhibited a significant decrease in individuals with mild DR across both retinal layers, and also in diabetes mellitus without DR (no DR) within DVP. EPD showed a significant increase in mild DR cases compared to HCs, except for the foveal region in SVP; however, EPN did not demonstrate a significant difference among the three groups. Increases in both FVSC and FVLR were significant across all areas and in both layers of the retina. Notably, these metrics showed more pronounced differentiation in the DVP than the SVP.

**Conclusions:**

Foveal BND and BNN in DVP reveal vascular alterations indicative of preclinical DR. Indicators such as EPD, FVSC, and FVLR in DVP correlate with early DR changes and are useful for its early detection. These initial findings demonstrate the potential and benefits of these quantitative OCTA indices for delineating DR-associated alterations in the retinal microvasculature, indicating their potential clinical utility for improved DR screening.

Diabetic retinopathy (DR), a prevalent severe complication of diabetes mellitus, impacts around three-quarters of diabetics 15 years post-diagnosis.^1^ It is a primary contributor to vision loss and blindness among individuals aged 20 to 74 globally.^2^ Forecasts suggest the number of patients with DR could surpass 160 million by 2045.^3^ Left untreated, DR may progress to serious conditions, such as vitreous hemorrhage, diabetic macular edema, and tractional retinal detachment, potentially resulting in blindness.^4^^,^^5^ Many patients with DR show no symptoms until the disease has advanced significantly.^6^ Therefore, early screening plays a crucial role in timely identifying pathologic signs, aiming to halt the progression of vision function declining and lower the risk of blindness.^7^

Color fundus imaging and ophthalmoscopy are recognized as the benchmark for diagnosing and staging DR, with the initial indicators often being microaneurysms observed in the retina.^8^^–^^10^ However, it is acknowledged that microvascular deterioration occurs prior to the visible manifestation of retinopathy.^11^ While fluorescein angiography (FA) continues to be a critical method for assessing retinal vascular abnormalities and a key diagnostic procedure for DR, its use is limited due to its invasive nature and its inability to effectively differentiate between signals at varying depths of the retina.^12^ Consequently, FA is not typically recommended as a screening method for patients in the early stages of DR.^1^

Over the last decade, optical coherence tomography angiography (OCTA), a functional extension of optical coherence tomography (OCT), has become an increasingly valuable tool for examining ocular microvasculature.^13^^,^^14^ In contrast to FA, OCTA offers a noninvasive approach, utilizing the motion of blood cells instead of exogenous dyes to generate angiograms.^12^ This is achieved by signal decorrelation between consecutive OCT B-scans.^13^ OCTA is particularly effective in measuring minute changes in capillaries caused by retinal diseases, offering detailed, layer-specific images of the retina, including the superficial and deep vascular layers. Its ability to provide high-resolution, depth-resolved views makes OCTA ideal for investigating vascular changes in the preclinical and initial stages of DR.^12^

In recent studies, researchers have explored quantitative OCTA indicators like perfused vascular density (VD), the foveal avascular zone (FAZ) area, and fractal dimension (FD) for assessing DR. VD measures blood vessel coverage; FAZ denotes a capillary-free zone in the macula, and FD assesses complexity across scales. Despite their potential, findings regarding these indicators vary significantly. Studies commonly report diminished VD in DR^15^^–^^17^ or diabetes mellitus without DR (no DR),^18^^,^^19^ comparing with healthy controls (HCs). Conversely, other research shows unchanged^20^ or even increased^21^ VD in patients with no DR. Similarly, discrepancies exist regarding the FAZ area, with some identifying enlargement in patients with DR,^15^^,^^22^^,^^23^ whereas others find no significant difference.^24^^–^^26^ The situation is akin to FD, associated with DR reduction by several studies,^27^^–^^30^ yet some indicate increased FD in early DR,^31^^–^^33^ or no relation at all.^34^ The inconsistent findings regarding these traditional indicators underscore not their limitations but the complex nature of DR and the multifaceted way it affects retinal microvasculature. These observations suggest that a broader array of metrics might provide a more comprehensive evaluation of DR's impact.

Therefore, this study focuses on an in-depth examination of the superficial vascular plexus (SVP) and deep vascular plexus (DVP) of the retina in individuals with no DR and those with mild DR, using OCTA. We introduced novel OCTA metrics to evaluate branching patterns and fragmentation features in retinal vasculature, including branch node number (BNN), branch node density (BND), end point number (EPN), end point density (EPD), fragmented vessel segment count (FVSC), and fragmented vascular length ratio (FVLR).

## Methods

### Study Population

This investigation incorporated 50 eyes from the HC group, 24 eyes from the no DR group, and 25 eyes from the mild DR group. Within the context of this study, mild DR refers to early mild nonproliferative DR (NPDR), whereas no DR denotes individuals with diabetic mellitus who do not have DR. Participants were recruited from the First Hospital of Qinhuangdao, and informed consent was secured from all subjects. This research adhered to the ethical guidelines of the Declaration of Helsinki and was approved by the Institutional Review Board of the First Hospital of Qinhuangdao and the Ethics Committee of Northeastern University Bio and Medical (NEU-EC-2023B008S). Eligibility criteria included: (1) a best-corrected visual acuity (BCVA) of 20/20 or better, age above 18 years, and being either healthy or diagnosed with type 2 diabetes mellitus by an endocrinologist; (2) participants with mild DR were limited to those exhibiting no more than mild NPDR; (3) absence of other ocular diseases or significant media opacity, no prior intraocular treatments (including laser therapy, intravitreal injections, cataract surgery, or vitreoretinal surgery), and a refractive error of less than 4 diopters; and (4) exclusion of individuals with ischemic heart disease, hypertension, or neurodegenerative disease.^27^^,^^35^^,^^36^

### OCTA Image Acquisition and Region Partitioning

**Figure 1.**
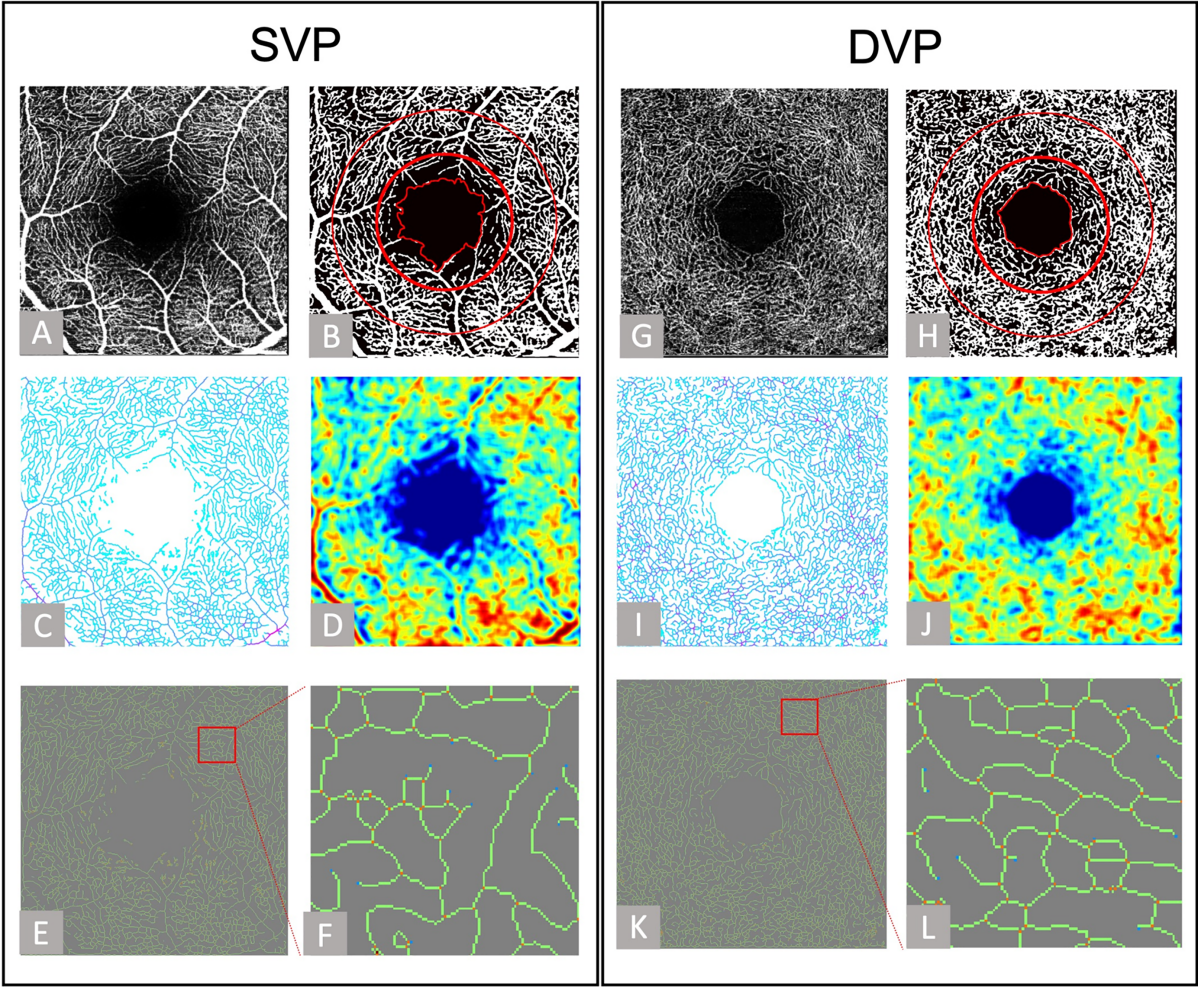
Overview of data processing procedures of SVP and DVP. Original OCTA image (**A** and **G**), binarized vascular image with FAZ boundary, 1.5 mm, and 2.5 mm circles highlighted in red (**B** and **H**), Vascular skeleton image (**C** and **I**), VD image (**D** and **J**), vascular skeleton image with vessel branch terminals labeled and enlarged view of the red box in **E** and **K** (**F** and **L**). In Figures **E**, **F**, **K**, and **L**, orange dots represent branch nodes and blue dots represent endpoints.

Retinal images via OCTA were captured using the Spectralis OCT system (Heidelberg, Germany). For each subject, both eyes underwent imaging with a scan comprising 512 B-scan clusters, each repeated 4 times, with every B-scan comprising 512 A-scans. The resultant 3D dataset spanned covering an area of 3 × 3 mm^2^. The OCTA software, specifically the Heyex Software version 1.9.201.0 from Heidelberg Engineering, Germany, was utilized to automatically segment the volumetric OCTA data into images of the SVP and DVP. The SVP is mainly found within the nerve fiber layer or the ganglion cell layer and the DVP is located predominantly within the inner nuclear layer. For analytical purposes, two concentric circles centered on the fovea's midpoint were drawn with diameters of 1.5 mm and 2.5 mm.^37^ The area within the boundary of the FAZ and the inner circle was defined as the fovea area, whereas the space between the two circles was identified as the parafovea area, as illustrated in Figure 1.

### FAZ Extraction

The analysis of the fovea region necessitates the exclusion of FAZ, making the detection and extraction of the FAZ from the OCTA image essential for the accurate measurement of vascular metrics. This study adopted a method integrating an adaptive watershed transformation with an active contour algorithm for FAZ delineation, as previously described.^38^ Initially, the OCTA vascular images were converted into topographic representations through distance transformation, where each point's value signifies the minimal distance to the nearest vascular pixel. Given that the FAZ center is the most distant from vascular pixels, it exhibits the highest distance value on this map. Subsequently, the topographic map was inverted, and a watershed algorithm was applied to segment it into distinct areas, with an adaptive technique utilized to mitigate issues of over-segmentation. The FAZ, being the most prominent “reservoir,” was thus readily identified. Further refinement of FAZ boundaries was achieved using an active contour algorithm, enhancing the precision of the extraction process.

### Vessel Binarization

Prior to the assessment of retinal vascular metrics in this study, vessel binarization was conducted. The “Local Adaptive Region Growing” algorithm, an enhancement of conventional region-growing methods and previously described,^39^ was utilized for vessel segmentation. The advantage of this algorithm is its ability to adaptively adjust the discrimination criteria based on the characteristics of target and background pixels within local regions. This adaptability ensures accurate segmentation by preventing the misidentification of the background as vessels in situations where both the background and vessel signal intensities are high, and by still being able to identify faint vessel signals when both are low. It avoids the premature halting of segmentation in areas with weak vascular signals and negates errors induced by high background signals. The resultant binarized OCT angiograms, as depicted in Figures 1B and 1H, clearly differentiate blood vessels, marked with a pixel value of 1, from the background, which is assigned a pixel value of 0.

### Vessel Skeletonization

The vascular skeleton was extracted from the binarized vascular image. In this study, a morphology-based thinning algorithm was used to extract the vascular skeleton.^40^ This algorithm iteratively removes boundary pixels from the binary image until the structure in the image is reduced to a skeleton with a width of one pixel. The skeleton extraction process includes the following steps: (a) identify boundary pixels in the image with certain structural characteristics; (b) through multiple iterations, remove these boundary pixels in a way that the structure shrinks while maintaining connectivity; and (c) the final output is a skeleton representation of the input image’s structure, with the features preserved in lines that are one pixel wide. To simultaneously reflect the vessel diameter, we applied a distance transform to the binarized vascular image. After the distance transform, each pixel value in the image represents the shortest distance from that point to the vessel boundary, which corresponds to the vessel radius. By pointwise multiplying the distance-transformed images with the vascular skeleton image, we obtained vascular skeleton images with diameter information, as shown in Figures 1C and 1I. For robustness testing of the vascular skeletonization method across OCTA images of varying quality, please refer to the Supplementary Materials (Supplementary Figs. S1, S2, S3).

### Quantification of VL, VAD, and VD

In this study, three crucial vascular parameters were measured from the binarized OCTA images: vascular length (VL), vascular average diameter (VAD), and VD.

VL: The process involved extracting the skeleton from segmented vessel images by eliminating pixels of the blood vessel not aligned with the central axis, as illustrated in Figures 1C and 1I. This skeletal representation corresponds to the VL of the vessels.

VAD: To calculate VAD, a distance transformation was applied to the binarized vessel images. This transformation assigns pixel values based on the shortest distance to the vessel's boundary, essentially representing the vessel's radius at its center. By doubling these values (to derive the diameter) and integrating them with the vessel skeleton, we produce an image where each pixel's value now represents the diameter of the vessel at that point. The average of these diameters across all vessels yields the VAD.

VD: Defined as the proportion of blood vessel pixels to the overall pixel count in the angiogram, VD measures the vascular coverage within the whole image. Local vascular density, defined as the vascular density within a localized area. For each pixel, the vascular density is calculated within a 30 × 30 pixel local region centered on that pixel, and the resulting value is assigned to the corresponding pixel. Once all pixels have been processed, a local vascular density map is generated. Representative figures are shown in Figures 1D and 1J.

### Identification of Vascular End Point and Branch Point

Within the skeletal map of the vessels, the pixels were classified into three distinct groups based on their connectivity to vessel branches: pixels connected to a single vessel branch were designated as end points, those linked to three or more branches were identified as branch nodes, and pixels connecting two branches were labeled as ordinary vessel pixels. These classifications were visually marked, with their representations showcased in Figures 1E and 1K. BNN is determined by counting the total orange dots within a specified area, whereas BND is calculated by dividing the BNN by the VL. Similarly, EPN tallies the total blue dots, and EPD is computed by dividing the EPN by VL.

### Quantification of Fragmented Vessels

The identification and quantification of “fragmented vessels” involved pinpointing all isolated vascular segments within the vascular skeleton image: specifically, those segments not connected to other vascular pixels. We measured the length of these individual segments, defining vessels as fragmented if their lengths fell below a predetermined threshold, namely, a specified multiple of the VAD. This approach to defining fragmented vessels, by tying segment length to VAD, aims to adjust for differences between individuals that may arise from variations in image area or resolution.

The selection of the VAD multiplier is pivotal for accurately analyzing fragmented vessel parameters. To this end, we examined a broad spectrum of multipliers, ranging from 2 times VAD up to 100 times VAD, to identify the most effective for classifying vascular fragmentation. Our analysis, detailed in the Supplementary Materials (Supplementary Fig. S4; Table S1), concluded that a multiplier of 30 times VAD optimized classification accuracy.

Building on this, we introduced two innovative metrics for the assessment of vascular fragmentation: FVSC, which quantifies the number of fragmented vessels, and FVLR, the ratio of the total length of fragmented vessels to the overall VL.

### Statistical Analysis

Statistical analyses were performed utilizing GraphPad Prism software (Graph-Pad Software Inc., San Diego, CA, USA), with the results presented as mean ± standard deviation (SD). The comparison between two distinct groups was executed through an unpaired two-tailed Student's *t*-test, considering a *P* value below 0.05 deemed indicative of statistical significance.

## Results

**Figure 2.**
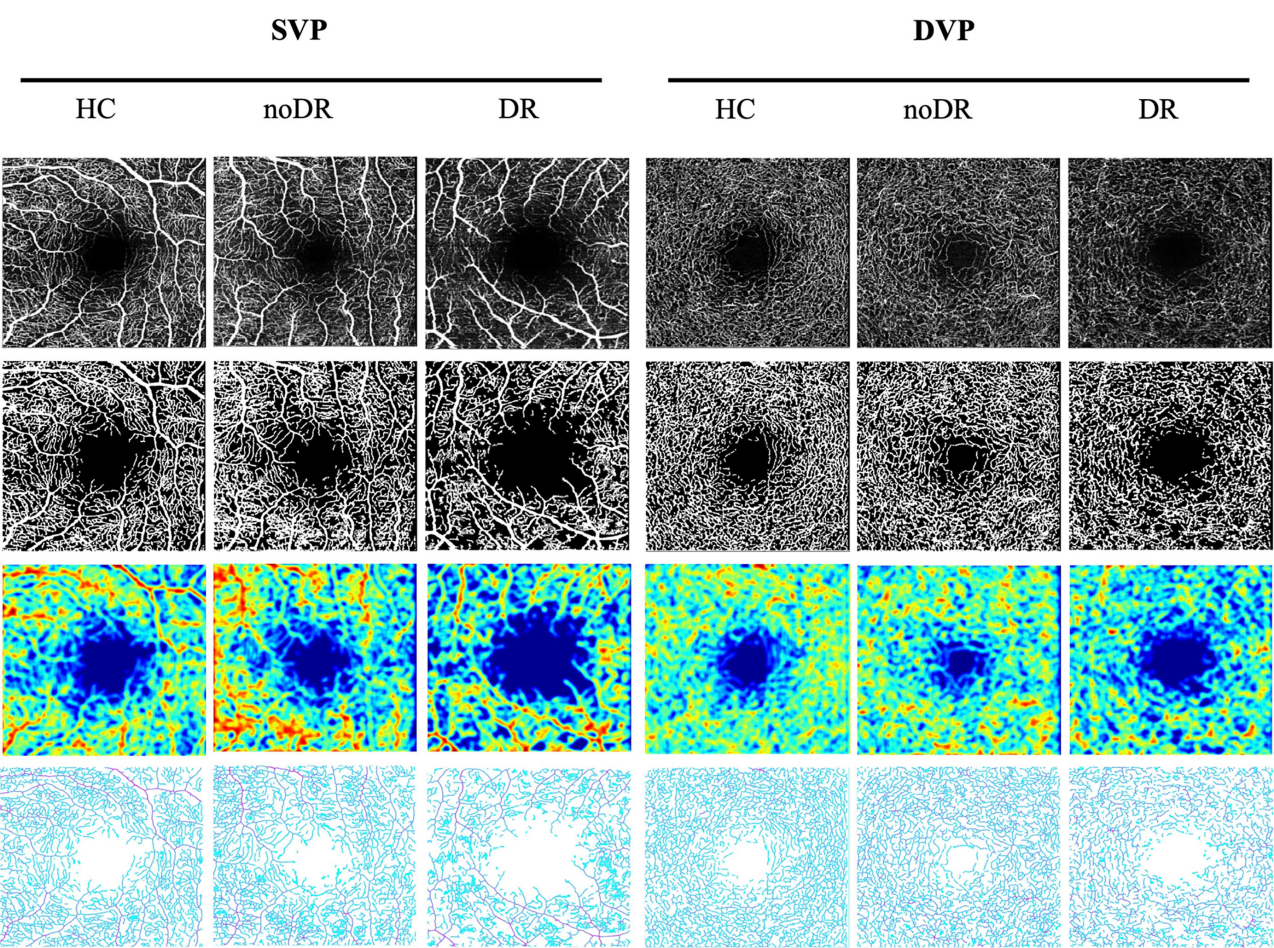
Representative OCTA images in a 3 × 3-mm area around the fovea. The first row: OCTA images; The second row: binarized images; The third row: VD images; The Last row: vascular skeleton images.

**Figure 3.**
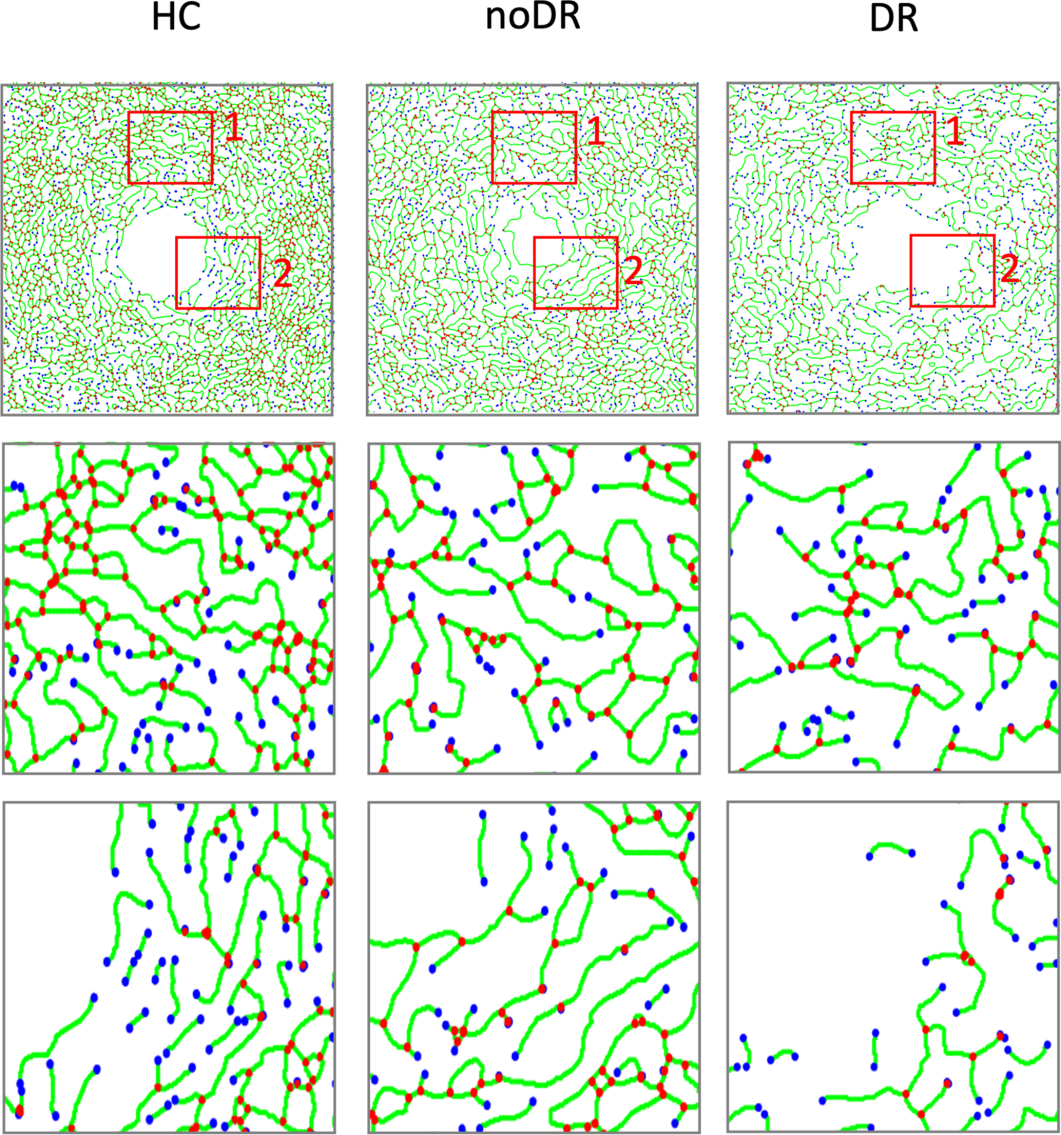
Representative processed OCTA images revealing branch nodes and endpoints in HC, noDR, and mild DR groups in DVP. The first row shows the original branch node- and endpoint-labeled images of three groups. The second and the third rows show the enlarged images of regions 1 and 2, respectively, from the corresponding 3 × 3 mm images. Branch nodes are marked with red dots and endpoints are labelled with blue dots.

This study analyzed 99 eyes from 55 participants (28 men and 27 women), without observing any gender-related differences in blood vessel density. Furthermore, no significant variations were noted in age or axial length across the three groups of subjects. Figure 2 displays representative OCTA images, alongside their binarized counterparts, perfusion maps, and skeletonized images for the SVP and DVP of HCs, no DR, and mild DR groups. Figure 3 illustrates vascular skeleton images with branch nodes marked in red and end points marked in blue, showcasing typical examples from each group.

**Figure 4.**
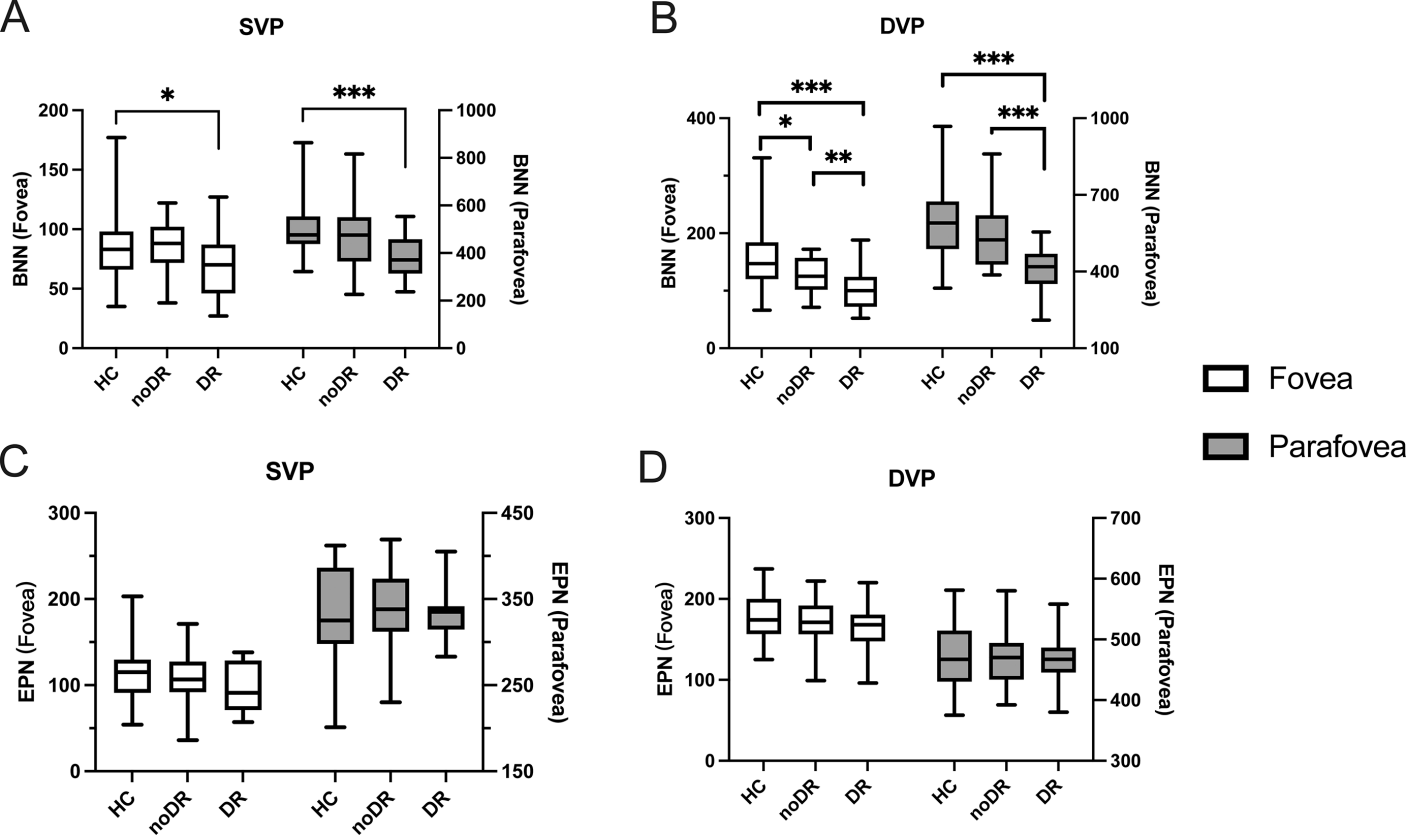
BNN and EPN measured in fovea and parafovea areas in SVP or DVP layer of retinal OCTA images acquired from three groups. BNN (**A** and **B**) and EPN (**C** and **D**) in the fovea (left Y axis) and parafovea (right Y axis) in SVP (left column) and DVP (right column) were calculated and compared among HC, noDR, and mild DR groups. Data were expressed as mean ± SD; Significant, * < 0.05, ** < 0.01, *** < 0.001. BNN, branch node number; EPN, endpoint number.

In both the fovea and parafovea areas, BNN exhibited a reduction in the mild DR group when compared to HCs (Figs. 4A, 4B). This decline was notably more pronounced in DVP (see Fig. 4B). Specifically, BNN was significantly lower in the mild DR group than in the HC group (*P* < 0.001), and also when compared to the no DR group (*P* < 0.01) in both regions, as shown in Figure 4B. Additionally, a significant decrease in BNN was observed in the no DR group relative to the HC group in the fovea region of the DVP (*P* < 0.05), highlighted in Figure 4B. Conversely, EPN showed no significant differences across the three groups in either SVP or DVP (Figs. 4C, 4D). Detailed quantitative and statistical analyses can be seen in Tables 1 and 2.

**Table 1.** Quantitative Analysis for Each Study Group of all Parameters

	HC	No DR	DR
	50 Eyes	22 Eyes	25 Eyes
**BNN**			
DVP			
Fovea	157.844 ± 52.759	128.273 ± 31.021	105.240 ± 39.340
Parafovea	595.578 ± 143.545	544.364 ± 129.974	413.640 ± 86.860
SVP			
Fovea	88.000 ± 33.767	84.318 ± 22.825	68.440 ± 28.918
Parafovea	503.511 ± 107.378	471.591 ± 166.847	389.880 ± 89.509
**BND %**			
DVP			
Fovea	2.584 ± 0.436	2.324 ± 0.362	2.143 ± 0.365
Parafovea	3.534 ± 0.454	3.411 ± 0.425	2.924 ± 0.350
SVP			
Fovea	2.150 ± 0.474	2.202 ± 0.419	1.881 ± 0.372
Parafovea	3.202 ± 0.361	3.164 ± 0.563	2.820 ± 0.345
**EPN**			
DVP			
Fovea	177.756 ± 29.306	171.000 ± 30.742	165.400 ± 29.052
Parafovea	472.089 ± 53.446	466.182 ± 47.099	466.200 ± 39.731
SVP			
Fovea	111.2 ± 29.923	108.273 ± 31.342	97.160 ± 28.291
Parafovea	337.533 ± 43.216	330.000 ± 59.237	331.200 ± 26.593
**EPD %**			
DVP			
Fovea	3.016 ± 0.467	3.147 ± 0.537	3.534 ± 0.614
Parafovea	2.878 ± 0.478	3.009 ± 0.537	3.367 ± 0.430
SVP			
Fovea	2.782 ± 0.447	2.780 ± 0.339	2.803 ± 0.450
Parafovea	2.195 ± 0.393	2.327 ± 0.446	2.453 ± 0.312
**FVSC**			
DVP			
Fovea	33.156 ± 10.408	36.227 ± 10.355	42.760 ± 11.322
Parafovea	57.400 ± 23.840	66.682 ± 25.376	84.920 ± 24.624
**FVLR %**			
DVP			
Fovea	13.567 ± 6.385	15.050 ± 5.524	22.207 ± 8.555
Parafovea	7.276 ± 3.916	8.554 ± 4.347	12.337 ± 5.627

Values are reported as mean ± SD.

**Table 2.** Statistical Analysis for Each Study Group of all Parameters

	BNN	BND	EPN	EPD	FVSC	FVLR
**HC v** **ersu** **s no** **DR**						
DVP						
Fovea	0.032*	0.020*	0.518	0.377	0.151	0.297
Parafovea	0.140	0.239	0.718	0.279	0.163	0.310
SVP						
Fovea	0.793	0.538	0.748	0.929	N/A	N/A
Parafovea	0.390	0.858	0.650	0.219	N/A	N/A
**HC v** **ersu** **s DR**						
DVP						
Fovea	<0.001***	<0.001***	0.201	<0.001***	0.001***	<0.001***
Parafovea	<0.001***	<0.001***	0.862	<0.001***	<0.001***	<0.001***
SVP						
Fovea	0.016*	0.009**	0.112	0.882	N/A	N/A
Parafovea	<0.001***	<0.001***	0.460	0.018	N/A	N/A
**No** **DR versus DR**						
DVP						
Fovea	0.020	0.099	0.469	0.029	0.056	0.003**
Parafovea	<0.001***	<0.001***	>0.999	0.023	0.031*	0.023*
SVP						
Fovea	0.032*	0.007**	0.176	0.808	N/A	N/A
Parafovea	0.071	0.028*	0.946	0.335	N/A	N/A

Significant, * < 0.05, ** < 0.01, *** < 0.001.

**Figure 5.**
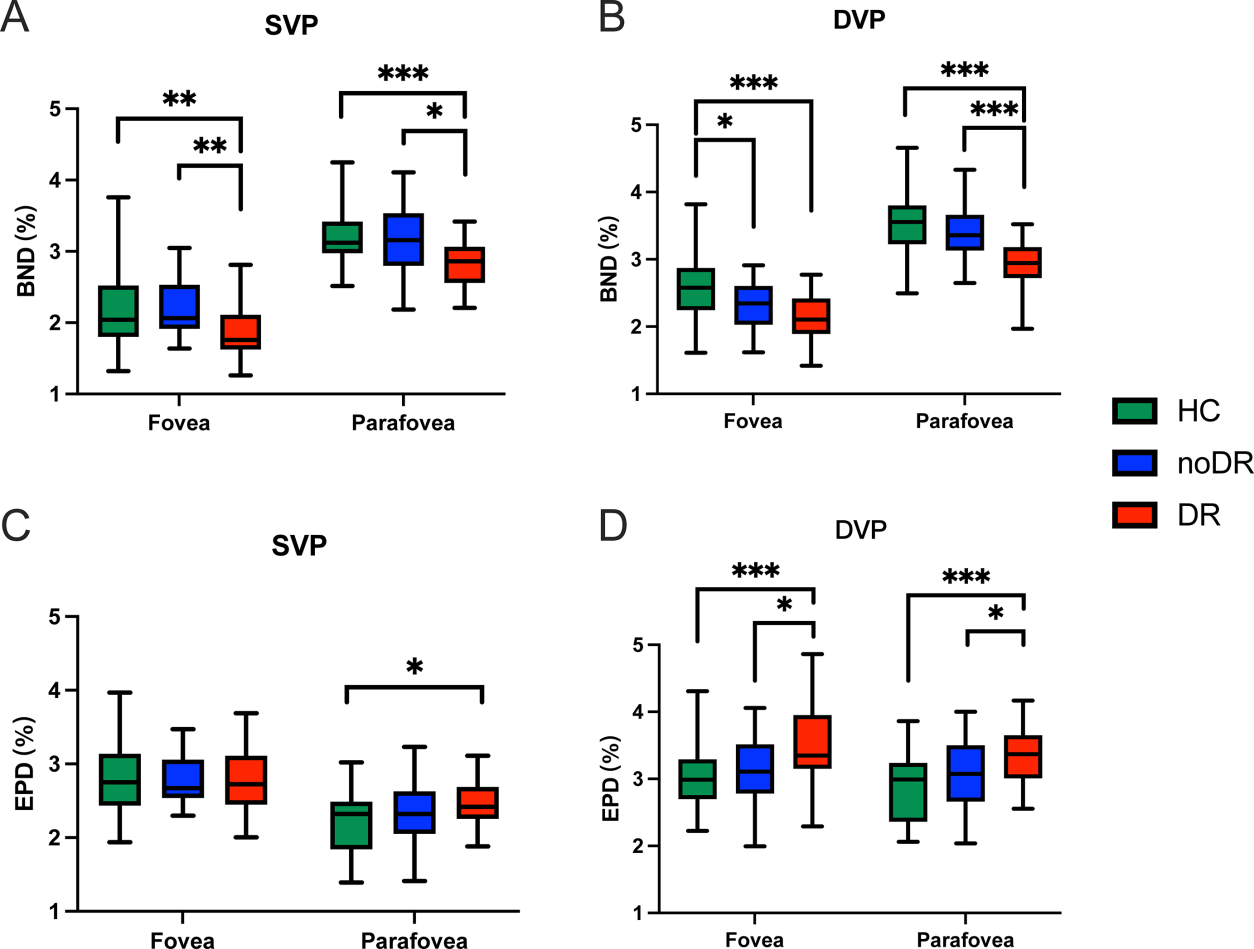
BND and EPD measured in fovea and parafovea areas in SVP or DVP layer of retinal OCTA images acquired from three groups. BND (**A** and **B**) and EPD (**C** and **D**) in fovea and parafovea in SVP (left column) and DVP (right column) were calculated and compared among HC, noDR, and mild DR groups. Data were expressed as mean ± S.D.; Significant, * < 0.05, ** < 0.01, *** < 0.001. BND, branch node density; EPD, endpoint density.

To evaluate the occurrences of BNN and the frequency of EPN, the counts per millimeter of VL were utilized as measures for BND and EPD, respectively. As depicted in Figures 5A and 5B, comparative analysis revealed that BND was significantly reduced in the mild DR group relative to HCs across both SVP and DVP, with a pronounced decline observed from the no DR to the mild DR condition. Notably, statistical analysis confirmed the ability to distinguish the no DR group from HCs with significance (*P* < 0.05; see Fig. 5B).

In contrast, EPD within SVP exhibited minimal variance among the groups, except for a slight but statistically significant elevation in the mild DR group compared to the HCs within the parafovea region (Fig. 5C). Furthermore, within the DVP, the mild DR group demonstrated a significant increase in EPD in both the fovea and parafovea regions when compared to both the HCs (*P* < 0.001) and the no DR (*P* < 0.05; Fig. 5D). Detailed quantitative and statistical analyses can be seen in Tables 1 and 2.

**Figure 6.**
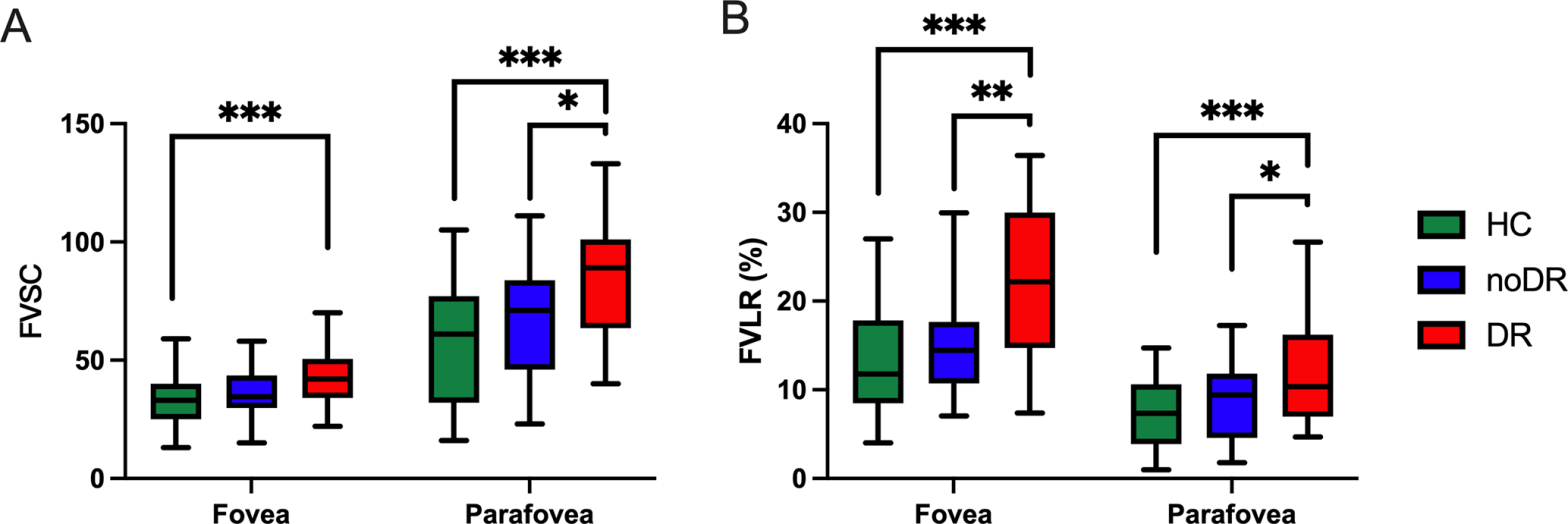
FVSC and FVLR measured in fovea and parafovea areas in the DVP layer of retinal OCTA images acquired from three groups. FVSC (**A**) and FVLR (**B**) in fovea and parafovea were calculated and compared among HC, noDR, and mild DR groups. Data were expressed as mean ± SD; Significant, * < 0.05, ** < 0.01, *** < 0.001. FVSC, fragmented vessel segment count; FVLR, fragmented vascular length ratio.

Building on earlier findings, it was observed that both BND and EPD exhibit significant changes primarily within the DVP rather than in the SVP. Consequently, the analysis of vascular fragmentation was exclusively focused on the DVP. FVSC, quantifying the total count of fragmented vessels, is significantly elevated in the mild DR group compared to the HCs within both the fovea and parafovea regions of DVP, as illustrated in Figure 6A. Notably, in the parafovea area, a marked distinction was also observed between the mild DR and the no DR groups.

Furthermore, FVLR, representing the ratio of the cumulative length of fragmented vessels to VL, was found to escalate with the disease's progression, showing a significant variance in the early clinically manifested mild DR stage. This escalation in FVLR was particularly pronounced from the no DR to mild DR stages, with significant differences noted between these groups in both the fovea and parafovea regions of DVP (Fig. 6B). Detailed quantitative and statistical analyses of the two parameters can be found in Tables 1 and 2.

## Discussion

In our study, we used six novel quantitative OCTA metrics to delve into the early vascular changes associated with DR, specifically targeting branch pattern and vessel fragmentation analyses in the retinal vasculature among three distinct cohorts: HCs, no DR, and mild DR. The analysis of branching patterns was conducted through parameters such as BNN, BND, EPN, and EPD, whereas the fragmentation analysis utilized metrics including FVSC and FVLR. Through these metrics, we aimed to identify easily overlooked and previously unexplored aspects of alterations within the retinal microvascular network and to bolster early screening and diagnostic efforts for this condition.

BNN refers to the total count of points within the analyzed area of retinal vasculature where a single blood vessel bifurcates, or branches, into two or more vessels. BNN is indicative of the complexity and connectivity of the retinal vascular network. BND is a measure of the branch nodes per millimeter (mm) of the vessel. It quantifies how densely populated the retinal area is with branching points. A higher BND can suggest a more complex vascular network, which may be necessary to meet to metabolic demands of the retinal tissue in that area. Our data show both BNN and BND decreased when comparing the mild DR group to the HC group in both the fovea and parafovea regions at SVP and DVP layers. As DR progresses, it can lead to damage to the retinal blood vessels. This damage may result in the loss or pruning of smaller vessel branches,^41^ leading to a decrease in BNNs. Consistently, Vujosevic et al. and Shi et al. both observed a significant reduction in a number of branches in the macular region in patients with early DR.^42^^,^^43^ This reduction in branching complexity may reflect a simplification of the retinal vascular network as the disease advances.^44^ The decrease in BND (BNN divided by vascular length) suggests that, as the disease progresses, the branching points become less frequent along the length of the blood vessels. This could be due to vessel constriction, damage, or pruning of smaller branches.^44^ The VL also decreases with the disease progression (data not shown), which indicates that a greater level of decrease in BNN can result in the reduction also observed in BND. It is to be noted, that among the novel parameters analyzed in the study, BNN and BND in the fovea region in the DVP were the only metrics to distinguish the patients with no DR from the HCs (*P* = 0.032 and *P* = 0.020, respectively). This suggests that small vessels and capillaries surrounding FAZ are more susceptible to disease progression,^45^^,^^46^ and BNN and BND in the fovea of the retinal DVP might be of great help in identifying the preclinical status of DR individuals and may have the potential as indicators of forewarning. FD, a metric used to indicate branching pattern fill space more thoroughly than a line, but less than a plane (usually lies between 1 and 2), measures the vasculature's global branching complexity. Previous work has shown a decrease in average FD in eyes with DR.^2^^,^^18^^,^^42^^,^^47^^–^^50^ Reduction in FD refers to less filling of space, this could be due to less branching or reduced vessel tortuosity.^41^ We did not find that the vessel tortuosity is significantly decreased, or previous work suggests it is more likely to be increased.^28^^,^^29^^,^^51^ In this study, the reduction of BNN and BND with disease progression, in accordance with the reduction of FD,^2^^,^^18^^,^^42^^,^^47^^–^^50^ especially more profoundly changes in the DVP,^2^ indicating that the reduction of small branching/vessel bifurcation might be the main reason for FD decrease. This is also true even by eliminating the aspect of reduction of VL because BND also decreases in patients with mild DR compared to the HCs. Parameters like BNN and BND not only provide new features (another aspect) of vasculature alteration but can be used to indicate the early stage of DR or even before the clinical manifestation.

EPN among the three groups was not significantly different, which could indicate that the disease process, at least until the mild DR stage, does not affect the branching to a degree that creates new terminal points or eradicates existing ones. On the other hand, an increase in EPD over disease progression indicates that the density of terminal points relative to the total vascular length is rising. This could be due to a reduction in the total VL observed by us and in previous studies,^26^^,^^29^^,^^52^ possibly due to vessel atrophy or pruning, which is a common phenomenon in various vascular diseases including DR.^53^^–^^55^ When VL decreases while the EPN remains constant, the result is an increase in EPD. The increase in EPD could be indicative of a compacting or consolidating vascular network where vessels are becoming shorter or disappearing,^56^^–^^58^ leading to a higher density of end points relative to the total vascular length. Another interpretation could be a compensatory mechanism where the vascular network attempts to maintain a certain level of functionality (evident from stable EPN) despite ongoing pathological changes that affect the VL. It is also noted that EPD changes are more profound in the DVP than in the SVP, suggesting small vessels and capillaries are more sensitive to hyperglycemia and susceptible to disease-induced alterations, which is also observed in other studies.^8^^,^^27^^,^^59^ This result may be explained by the distinctive vascular components between the SVP and DVP networks. The SVP contains relatively more large vessels and, therefore, may have a relatively slow response to diabetes.^60^^,^^61^ The vascular network in the deep retinal layers might be undergoing remodeling or reorganization to maintain tissue perfusion,^60^^,^^62^ which could lead to an increase in EPD as vessels become shorter or undergo pruning,^63^ thereby increasing the density of end points relative to the total vascular length.

Quantitative analysis reveals a progressive increase in FVSC and FVLR from HCs to those patients with no DR, and further in patients with mild DR. This trend highlights the primary manifestation of vascular damage as an increase in vessel fragmentation, marking the significant adverse impact of diabetes on the microvascular system. The FVLR measures the length of fragmented vessels as a proportion of the total vascular length. The increased FVLR indicates that the fragmentation is not just increasing in number (as would be reflected by FVSC) but is also spreading across more substantial lengths of the vessels. Although prior research has explored various aspects of vascular damage, to the best of our knowledge, there has been no specific focus on quantifying vessel fragmentation in the way our study does, which we believe represents a novel contribution to the field. “Fragmented vessels” could result from microvascular damage, inadequate vascular growth, and alterations in blood flow dynamics.^2^^,^^5^^,^^64^ Within the diabetic milieu, sustained hyperglycemia inflicts damage upon endothelial cells lining the vessels, compromising their normal growth and reparative capacities.^5^^,^^65^ Such detriment may precipitate the disruption or discontinuity of vascular structures, manifesting as “fragmented vessels” on retinal scans. As diabetes progresses, exacerbation of vascular damage could lead to an increased formation of these fragmented vessels. Furthermore, an increase in vascular resistance, contributing to uneven blood flow, can make vessels appear discontinuous or “fragmented” in OCTA images. This is compounded by fluctuations in blood flow velocity, which may affect the OCTA’s ability to detect blood flow consistently, causing some vascular segments to be undetected and thereby contributing to the appearance of fragmentation in imaging outputs. The absence of significant growth between healthy and diabetic cohorts might be attributed to the early stages of diabetes, where the extent of microvascular damage has yet to reach detectable levels in OCTA imagery (see Fig. 6). At this juncture, despite the initiation of vascular damage, the vessels’ intrinsic repair capabilities and compensatory mechanisms might temporarily offset the deleterious effects.^21^^,^^51^^,^^66^ A substantial difference is observed transitioning comparing patients with mild DR to HCs (*P* ≤ 0.001), indicating that the progression to DR is characterized by poor neovascularization and extensive microvascular damage, leading to increased isolation of vascular segments and a significant rise in the number of “fragmented vessels.”

Our findings suggest a paradigm shift toward incorporating these advanced metrics into routine clinical screenings, potentially enabling earlier intervention strategies. Future studies should further explore the integration of these OCTA metrics with clinical outcomes to validate their utility in DR management and screening protocols, considering the variability and potential limitations observed in sensitivity analyses across different OCTA studies. This study faces several limitations. First, the small dataset size, combined with the homogeneous sample population of Chinese patients with type 2 diabetes mellitus, potentially limits the applicability of our results across diverse ethnic groups. Furthermore, our reliance on OCTA metrics concentrated on the macula, compared with a smaller field of view than that offered by FA, could confine our insights into the vascular changes occurring in the peripheral retina during the initial stages of DR.

## Conclusions

In conclusion, our study reveals marked several changes in our developed metrics among HCs, patients with no DR, and patients with mild DR. This investigation enhances the granularity of retinal vascular assessments and elucidates the complex relationship between retinal vascular modifications and the diabetic milieu, thus refining our understanding of DR vascular pathogenesis. The outcomes of this research advocate for the integration of these advanced OCTA metrics into clinical protocols for the early detection and management of DR.

## Supplementary Material

Supplement 1

Supplement 2

Supplement 3

## References

[1] Agra CLD, Lira RPC, Pinheiro FG, Sa L, Filho V. Optical coherence tomography angiography: microvascular alterations in diabetic eyes without diabetic retinopathy. *Arq Bras Oftalmol**.* 2021; 84(2): 149–157. 33567012 10.5935/0004-2749.20210023 PMC12289254

[2] Chen Q, Ma QK, Wu CM, et al. Macular vascular fractal dimension in the deep capillary layer as an early indicator of microvascular loss for retinopathy in type 2 diabetic patients. *Invest Ophthalmol Vis Sci**.* 2017; 58(9): 3785–3794. 28744552 10.1167/iovs.17-21461

[3] Ogurtsova K, da Rocha Fernandes J, Huang Y, et al. IDF Diabetes Atlas: global estimates for the prevalence of diabetes for 2015 and 2040. *Diabetes Res Clin Pract**.* 2017; 128: 40–50. 28437734 10.1016/j.diabres.2017.03.024

[4] Tang J, Kern TS. Inflammation in diabetic retinopathy. *Prog Retin Eye Res**.* 2011; 5(30): 343–358. 10.1016/j.preteyeres.2011.05.002 PMC3433044 21635964

[5] Al-Kharashi AS . Role of oxidative stress, inflammation, hypoxia and angiogenesis in the development of diabetic retinopathy. *Saudi J Ophthalmol**.* 2018; 32(4): 318–323. 30581303 10.1016/j.sjopt.2018.05.002 PMC6300752

[6] Lawrenson J, Bourmpaki E, Bunce C, et al. Trends in diabetic retinopathy screening attendance and associations with vision impairment attributable to diabetes in a large nationwide cohort. *Diabet Med**.* 2021; 38(4): e14425. 33064854 10.1111/dme.14425 PMC8048647

[7] Stefánsson E, Bek T, Porta M, Larsen N, Kristinsson JK, Agardh E. Screening and prevention of diabetic blindness. *Acta Ophthalmol Scand**.* 2000; 78(4): 374–385. 10990036 10.1034/j.1600-0420.2000.078004374.x

[8] Li Rudvan AL, Can ME, Efe FK, Keskin M, Beyan E. Evaluation of retinal microvascular changes in patients with prediabetes. *Niger J Clin Pract**.* 2021; 24(6): 911–918. 34121741 10.4103/njcp.njcp_193_20

[9] Lupidi M, Coscas G, Coscas F, et al. Retinal microvasculature in nonproliferative diabetic retinopathy: automated quantitative optical coherence tomography angiography assessment. *Ophthalmic Res**.* 2017; 58(3): 131–141. 28538221 10.1159/000471885

[10] Friedenwald J, Day R. The vascular lesions of diabetic retinopathy. *Bull Johns Hopkins Hosp**.* 1950; 86(4): 253–254. 15411556

[11] Ulutas HG, Guclu M, Aslanci ME, Karatas G. The relationship between carotid intima-media thickness and microvascular changes in retinal zones and optic disc in patients with type 1 diabetes mellitus. *Eur J Ophthalmol**.* 2022; 32(4): 2328–2337. 34851200 10.1177/11206721211064024

[12] Chen SY, Moult EM, Zangwill LDM, Weinreb RN, Fujimoto JG. Geometric perfusion deficits: a novel OCT angiography biomarker for diabetic retinopathy based on oxygen diffusion. *Am J Ophthalmol**.* 2021; 222: 256–270. 32918905 10.1016/j.ajo.2020.09.007 PMC8015788

[13] De Carlo TE, Romano A, Waheed NK, Duker JS. A review of optical coherence tomography angiography (OCTA). *Int J Retina Vitreous**.* 2015; 1: 5. 27847598 10.1186/s40942-015-0005-8 PMC5066513

[14] De Carlo TE, Chin AT, Bonini MA, et al. Detection of microvascular changes in eyes of patients with diabetes but not clinical diabetic retinopathy using optical coherence tomography angiography. *Retina**.* 2015; 35(11): 2364–2370. 26469537 10.1097/IAE.0000000000000882

[15] Wang H, Liu XH, Hu XF, Xin H, Bao H, Yang S. Retinal and choroidal microvascular characterization and density changes in different stages of diabetic retinopathy eyes. *Front Med (Lausanne)**.* 2023; 10: 1186098. 37564040 10.3389/fmed.2023.1186098 PMC10411453

[16] Al-Sheikh M, Akil H, Sadda SR. Swept-source OCT imaging of the foveal avascular zone and macular capillary network in diabetic retinopathy. *Invest Ophthalmol Vis Sci**.* 2016; 57(8): 3907–3913. 27472076 10.1167/iovs.16-19570

[17] Tang FY, Chan EO, Sun ZH, et al. Clinically relevant factors associated with quantitative optical coherence tomography angiography metrics in deep capillary plexus in patients with diabetes. *Eye Vis (Lond)**.* 2020; 7(1): 7. 32025523 10.1186/s40662-019-0173-y PMC6996172

[18] Zahid S, Dolz-Marco R, Freund KB, et al. Fractal dimensional analysis of optical coherence tomography angiography in eyes with diabetic retinopathy. *Invest Ophthalmol Vis Sci**.* 2016; 57(11): 4940–4947. 27654421 10.1167/iovs.16-19656

[19] Carnevali A, Sacconi R, Corbelli E, et al. Optical coherence tomography angiography analysis of retinal vascular plexuses and choriocapillaris in patients with type 1 diabetes without diabetic retinopathy. *Acta Diabetol**.* 2017; 54(7): 695–702. 28474119 10.1007/s00592-017-0996-8

[20] Goudot MM, Sikorav A, Semoun O, et al. Parafoveal OCT angiography features in diabetic patients without clinical diabetic retinopathy: a qualitative and quantitative analysis. *J Ophthalmol**.* 2017; 2017: 8676091. 28761762 10.1155/2017/8676091 PMC5518527

[21] Rosen RB, Romo JSA, Krawitz BD, et al. Earliest evidence of preclinical diabetic retinopathy revealed using optical coherence tomography angiography perfused capillary density. *Am J Ophthalmol**.* 2019; 203: 103–115. 30689991 10.1016/j.ajo.2019.01.012 PMC6612596

[22] Um T, Seo EJ, Kim YJ, Yoon YH. Optical coherence tomography angiography findings of type 1 diabetic patients with diabetic retinopathy, in comparison with type 2 patients. *Graefes Arch Clin Exp Ophthalmol**.* 2020; 258(2): 281–288. 31832768 10.1007/s00417-019-04517-6

[23] Johannesen SK, Viken JN, Vergmann AS, Grauslund J. Optical coherence tomography angiography and microvascular changes in diabetic retinopathy: a systematic review. *Acta Ophthalmol**.* 2019; 97(1): 7–14. 30238633 10.1111/aos.13859

[24] Yang XY, Yi GG, Chen YX, et al. Optical coherence tomography angiography metrics in diabetes: focusing on diabetic retinopathy and carotid atherosclerosis. *Photodiagnosis Photodyn Ther**.* 2023; 44: 103799. 37696316 10.1016/j.pdpdt.2023.103799

[25] Simonett JM, Scarinci F, Picconi F, et al. Early microvascular retinal changes in optical coherence tomography angiography in patients with type 1 diabetes mellitus. *Acta Ophthalmol**.* 2017; 95(8): e751–e755. 28211261 10.1111/aos.13404

[26] Durbin MK, An L, Shemonski ND, et al. Quantification of retinal microvascular density in optical coherence tomographic angiography images in diabetic retinopathy. *JAMA Ophthalmol**.* 2017; 135(4): 370–376. 28301651 10.1001/jamaophthalmol.2017.0080 PMC5470403

[27] Wu Y, He MG, Huang WY, Wang W. Associations between retinal microvascular flow, geometry, and progression of diabetic retinopathy in type 2 diabetes: a 2-year longitudinal study. *Acta Diabetol**.* 2024; 61: 195–204. 37819475 10.1007/s00592-023-02194-w

[28] Liu JH, Chen SH, Xu ZY, et al. Macular vascular complexity analysis of diabetes mellitus by swept-source optical coherence tomographic angiography. *Ophthalmologica**.* 2023; 245(6): 538–545. 10.1159/000528073 36384762

[29] Wang W, Chen YP, Kun X, et al. Flow and geometrical alterations in retinal microvasculature correlated with the occurrence of diabetic retinopathy evidence from a longitudinal study. *Retina**.* 2022; 42(9): 1729–1736. 35502958 10.1097/IAE.0000000000003518

[30] Forster RB, Garcia ES, Sluiman AJ, et al. Retinal venular tortuosity and fractal dimension predict incident retinopathy in adults with type 2 diabetes: the Edinburgh Type 2 Diabetes Study. *Diabetologia**.* 2021; 64: 1103–1112. 33515071 10.1007/s00125-021-05388-5 PMC8012328

[31] Ţălu Ş, Călugăru DM, Lupaşcu CA. Characterisation of human non-proliferative diabetic retinopathy using the fractal analysis. *Int J Ophthalmol**.* 2015; 8(4): 770. 26309878 10.3980/j.issn.2222-3959.2015.04.23 PMC4539644

[32] Lim LS, Chee ML, Cheung CY, Wong TY. Retinal vessel geometry and the incidence and progression of diabetic retinopathy. *Invest Ophthalmol Vis Sci**.* 2017; 58(6): BIO200–BIO205. 28750414 10.1167/iovs.17-21699

[33] Cheung N, Rogers SL, Donaghue KC, Jenkins AJ, Tikellis G, Wong TY. Retinal arteriolar dilation predicts retinopathy in adolescents with type 1 diabetes. *Diabetes Care**.* 2008; 31(9): 1842–1846. 18523143 10.2337/dc08-0189 PMC2518356

[34] Klein R, Lee KE, Danforth L, et al. The relationship of retinal vessel geometric characteristics to the incidence and progression of diabetic retinopathy. *Ophthalmology**.* 2018; 125(11): 1784–1792. 29779685 10.1016/j.ophtha.2018.04.023 PMC6188797

[35] Han YQ, Wang XG, Sun G, et al. Quantitative evaluation of retinal microvascular abnormalities in patients with type 2 diabetes mellitus without clinical sign of diabetic retinopathy. *Transl Vis Sci Technol**.* 2022; 11(4): 20. 10.1167/tvst.11.4.20 PMC9034707 35446407

[36] Altinisik M, Kahraman NS, Kurt E, Mayali H, Kayikcioglu O. Quantitative analysis of early retinal vascular changes in type 2 diabetic patients without clinical retinopathy by optical coherence tomography angiography. *Int Ophthalmol**.* 2022; 42(2): 367–375. 35099665 10.1007/s10792-022-02230-8

[37] Kolb H, Fernandez E, Jones B, Nelson R, eds. Webvision: The organization of the retina and visual system (Internet). Salt Lake City, UT: University of Utah Health Sciences Center; 1995. 21413389

[38] Liu J, Yan S, Lu N, et al. Automatic segmentation of foveal avascular zone based on adaptive watershed algorithm in retinal optical coherence tomography angiography images. *J Innov Opt Health Sci**.* 2022; 15(01): 2242001.

[39] Yu Y, Zhang N, Xiang B, et al. In vivo characterization of cerebrovascular impairment induced by amyloid β peptide overload in glymphatic clearance system using swept-source optical coherence tomography. *Neurophotonics**.* 2023; 10(1): 015005. 36817752 10.1117/1.NPh.10.1.015005 PMC9933996

[40] Lee T-C, Kashyap RL, Chu C-N. Building skeleton models via 3-D medial surface axis thinning algorithms. *CVGIP: Graphical Models and Image Processing**.* 1994; 56(6): 462–478.

[41] Reif R, Qin J, An L, Zhi Z, Dziennis S, Wang R. Quantifying optical microangiography images obtained from a spectral domain optical coherence tomography system. *Int J Biomed Imaging**.* 2012; 2012: 509783. 22792084 10.1155/2012/509783 PMC3389716

[42] Vujosevic S, Toma C, Villani E, et al. Early detection of microvascular changes in patients with diabetes mellitus without and with diabetic retinopathy: comparison between different swept-source OCT-A instruments. *J Diabetes Res**.* 2019; 2019: 2547216. 31281849 10.1155/2019/2547216 PMC6594252

[43] Shi Y, Lin PY, Ruan YM, Lin CF, Hua SS, Li B. Quantitative analysis of early diabetic retinopathy based on optical coherence tomography angiography biological image. *World J Clin Cases**.* 2021; 9(25): 7365–7371. 34616803 10.12998/wjcc.v9.i25.7365 PMC8464476

[44] Kim AY, Chu ZD, Shahidzadeh A, Wang RKK, Puliafito CA, Kashani AH. Quantifying microvascular density and morphology in diabetic retinopathy using spectral-domain optical coherence tomography angiography. *Invest Ophthalmol Vis Sci**.* 2016; 57(9): OCT362–OCT370. 27409494 10.1167/iovs.15-18904 PMC4968771

[45] Amato A, Nadin F, Borghesan F, et al. Widefield optical coherence tomography angiography in diabetic retinopathy. *J Diabetes Res**.* 2020; 2020: 8855709. 33299892 10.1155/2020/8855709 PMC7707991

[46] Hirano T, Kitahara J, Toriyama Y, Kasamatsu H, Murata T, Sadda S. Quantifying vascular density and morphology using different swept-source optical coherence tomography angiographic scan patterns in diabetic retinopathy. *Br J Ophthalmol**.* 2019; 103(2): 216–221. 29706601 10.1136/bjophthalmol-2018-311942

[47] Tang FY, Ng DS, Lam A, et al. Determinants of quantitative optical coherence tomography angiography metrics in patients with diabetes. *Sci Rep**.* 2017; 7: 2575. Erratum in *Sci* *Rep*. 2018;8(1):7314. 29728691 10.1038/s41598-018-25619-x PMC5935703

[48] Bhardwaj S, Tsui E, Zahid S, et al. Value of fractal analysis of optical coherence tomography angiography in various stages of diabetic retinopathy. *Retina**.* 2018; 38(9): 1816–1823. 28723846 10.1097/IAE.0000000000001774

[49] Zhu TP, Ma JL, Li JY, et al. Multifractal and lacunarity analyses of microvascular morphology in eyes with diabetic retinopathy: a projection artifact resolved optical coherence tomography angiography study. *Microcirculation**.* 2019; 26(3): e12519. 30480851 10.1111/micc.12519

[50] Hashmi S, Lopez J, Chiu B, Sarrafpour S, Gupta A, Young J. Fractal dimension analysis of OCTA images of diabetic retinopathy using circular mass-radius method. *Ophthalmic Surg Lasers Imaging Retina**.* 2021; 52(3): 116–122. 34038685 10.3928/23258160-20210302-01

[51] Zhao Q, Wang CT, Meng LH, et al. Central and peripheral changes in the retina and choroid in patients with diabetes mellitus without clinical diabetic retinopathy assessed by ultra-wide-field optical coherence tomography angiography. *Front Public Health**.* 2023; 11: 1194320. 37383256 10.3389/fpubh.2023.1194320 PMC10293646

[52] Wang XG, Han YQ, Sun G, et al. Detection of the microvascular changes of diabetic retinopathy progression using optical coherence tomography angiography. *Transl Vis Sci Technolo**.* 2021; 10(7): 31. 10.1167/tvst.10.7.31 PMC8254014 34191017

[53] Hwang TS, Jia YL, Gao SS, et al. Optical coherence tomography angiography features of diabetic retinopathy. *Retina**.* 2015; 35(11): 2371–2376. 26308529 10.1097/IAE.0000000000000716 PMC4623938

[54] Hsiao CC, Yang CM, Yang CH, Ho TC, Lai TT, Hsieh YT. Correlations between visual acuity and macular microvasculature quantified with optical coherence tomography angiography in diabetic macular oedema. *Eye**.* 2020; 34(3): 544–552. 31406356 10.1038/s41433-019-0549-1 PMC7042332

[55] Shaikh NF, Vohra R, Balaji A, et al. Role of optical coherence tomography-angiography in diabetes mellitus: utility in diabetic retinopathy and a comparison with fluorescein angiography in vision threatening diabetic retinopathy. *Indian J Ophthalmol**.* 2021; 69(11): 3218–3224. 34708776 10.4103/ijo.IJO_1267_21 PMC8725072

[56] Konno A, Ishibazawa A, De Pretto L, Shimouchi A, Omae T, Song YS. Relationship between nonperfusion area from widefield optical coherence tomography angiography and macular vascular parameters in diabetic retinopathy. *Int Ophthalmol**.* 2023; 43: 4803–4814. 37851140 10.1007/s10792-023-02882-0 PMC10724328

[57] Kim K, Kim ES, Yu SY. Prediction of diabetic retinopathy severity using a combination of retinal neurodegeneration and capillary nonperfusion on optical coherence tomography angiography. *Retina**.* 2023; 43(8): 1291–1300. 37116460 10.1097/IAE.0000000000003820

[58] Kaizu Y, Nakao S, Arima M, et al. Capillary dropout is dominant in deep capillary plexus in early diabetic retinopathy in optical coherence tomography angiography. *Acta Ophthalmol**.* 2019; 97(5): E811–E812. 30690901 10.1111/aos.14041

[59] Kaoual H, Braham IZ, Boukari M, Zhioua R. Evaluation of the effect of the severity of diabetic retinopathy on microvascular abnormalities and vascular density using optical coherence tomography angiography. *Acta Diabetol**.* 2021; 58(12): 1683–1688. 34313844 10.1007/s00592-021-01774-y

[60] Ashraf M, Sampani K, Clermont A, et al. Vascular density of deep, intermediate and superficial vascular plexuses are differentially affected by diabetic retinopathy severity. *Invest Ophthalmol Vis Sci**.* 2020; 61(10): 61. 10.1167/iovs.61.10.53 PMC7463180 32866267

[61] Cabral D, Pereira T, Ledesma-Gil G, et al. Volume rendering of dense B-scan optical coherence tomography angiography to evaluate the connectivity of macular blood flow. *Invest Ophthalmol Vis Sci**.* 2020; 61(6): 44. 10.1167/iovs.61.6.44 PMC7415320 32561927

[62] Agemy SA, Scripsema NK, Shah CM, et al. Retinal vascular perfusion density mapping using optical coherence tomography angiography in normals and diabetic retinopathy patients. *Retina**.* 2015; 35(11): 2353–2363. 26465617 10.1097/IAE.0000000000000862

[63] Chen YJ, Khouri AS, Zarbin MA, Szirth BC. Early retinal microvascular abnormalities in young adults with type 1 diabetes mellitus without clinically evident diabetic retinopathy. *Retina**.* 2021; 41(7): 1478–1486. 33252580 10.1097/IAE.0000000000003047

[64] Fragiotta S, Costanzo E, Picconi F, et al. Progression biomarkers of microvascular and photoreceptor changes upon long-term evaluation in type 1 diabetes. *Invest Ophthalmol Vis Sci**.* 2023; 64(5): 23. 10.1167/iovs.64.5.23 PMC10214881 37227747

[65] Ferrara M, Loda A, Coco G, et al. Diabetic retinopathy: soluble and imaging ocular biomarkers. *J Clin Med**.* 2023; 12(3): 912. 36769560 10.3390/jcm12030912 PMC9917666

[66] Dadzie AK, Le DV, Abtahi M, et al. Normalized blood flow index in optical coherence tomography angiography provides a sensitive biomarker of early diabetic retinopathy. *Transl Vis Sci Technol**.* 2023; 12(4): 3. 10.1167/tvst.12.4.3 PMC10082385 37017960

