# Supplementary material for: Early Diabetic Retinopathy Evaluation With OCTA: A Study on Vascular Branching and Fragmentation
Source: Invest Ophthalmol Vis Sci. 2024 Dec 10;65(14):21. doi: 10.1167/iovs.65.14.21 (PMC11636659; doi:10.1167/iovs.65.14.21)
Supplement: Supplement 1 [file iovs-65-14-21_s001.pdf]

Supplementary Information

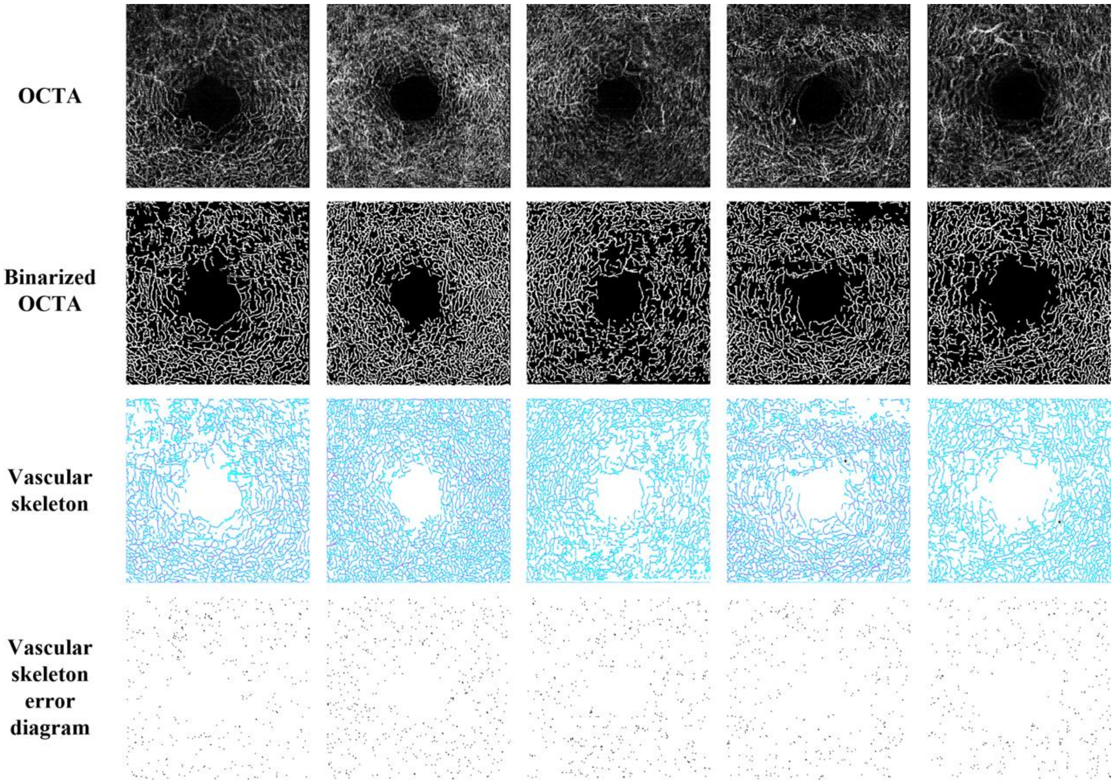

Figure S1, Representative vascular skeleton extraction results from low-quality OCTA vascular images.

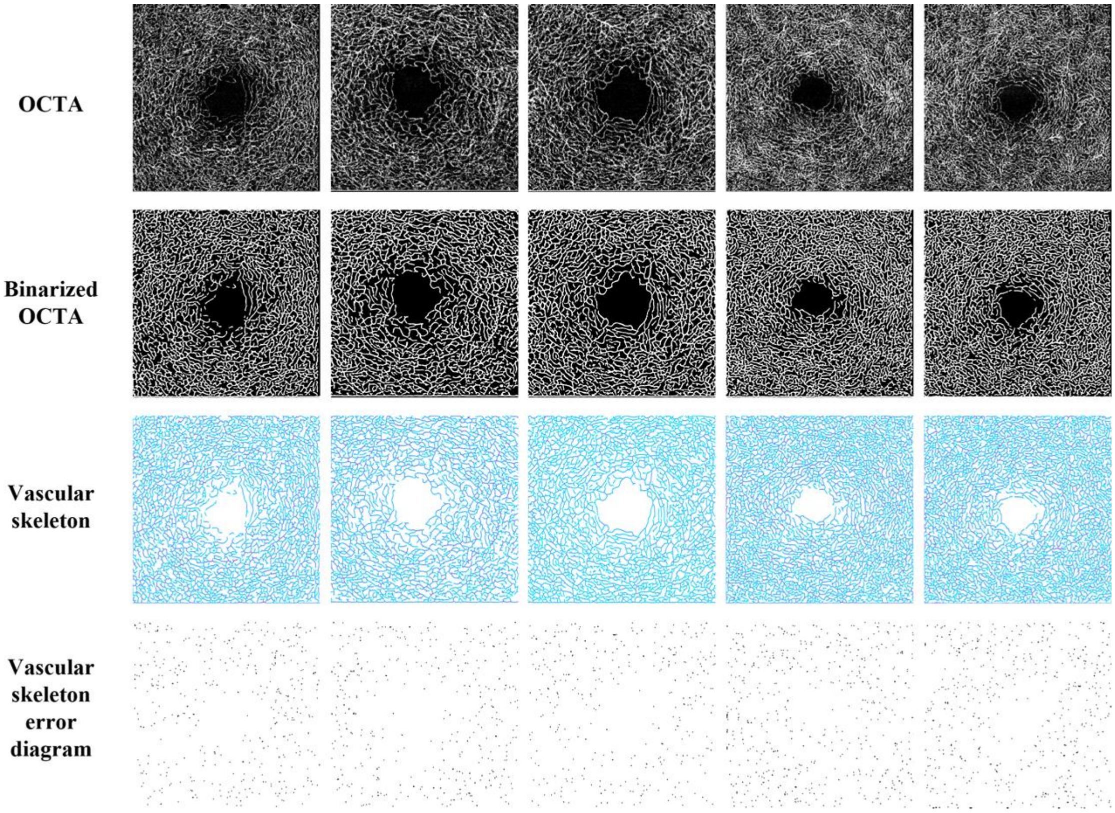

Figure S2, Representative vascular skeleton extraction results from high-quality OCTA vascular images.

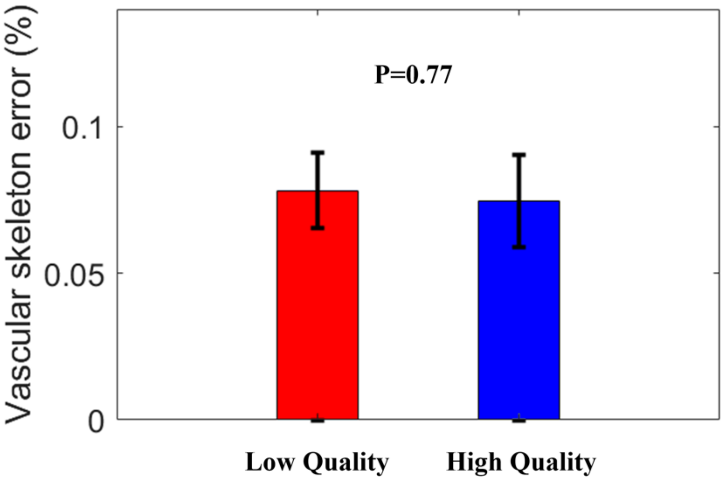

Figure S3, Comparison of extraction errors in vascular skeletons between low-quality and high-quality OCTA images.

Quantitative vascular fragmentation metrics rely on the defining of fragmented vessels. To establish an optimal threshold, the length under which is classified as fragmented, we used the diameter ratio method. This method is often used in medical research to analyze blood vessels or other structures. Specifically, we assessed the statistics of FVSC and FVLR for three groups of subjects in fovea and parafovea regions in DVP at thresholds between 5 to 100 times VAD. Figure S1 showed that both parameters of the three groups increased with increasing values of threshold. The minimum P value is required to best distinguish between HC and DR. We found that the minimum P values obtained were found at a threshold of 30 times the VAD (Table S1). Therefore, the vessels shorter than 30 times VAD were defined as fragmented vessels.

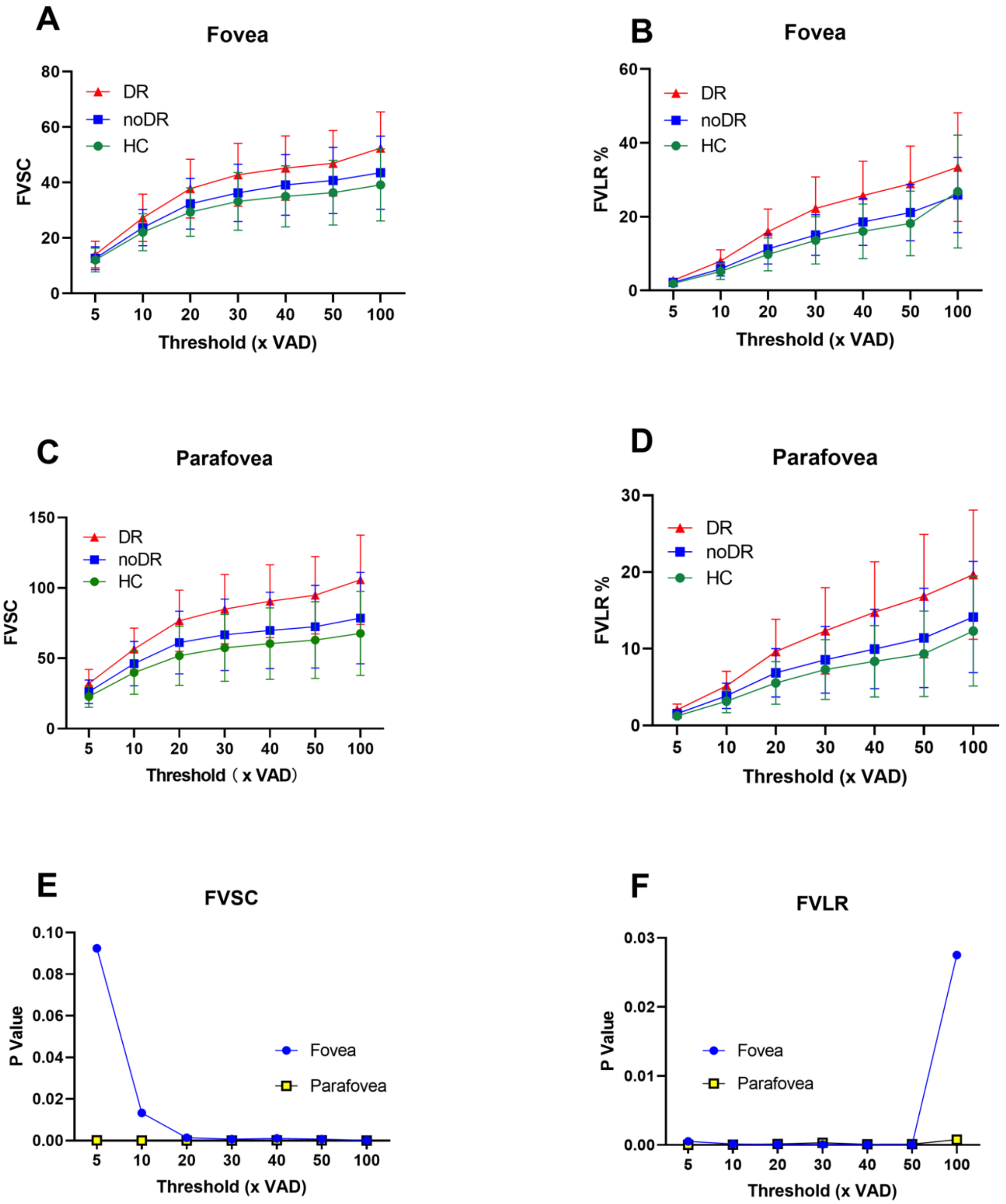

Figure S4, Statistics for FVSC and FVLR in DVP at different threshold values of HC, noDR, and DR. FVSC values are plotted against the threshold value (×VAD) for fovea (A) and parafovea (C) regions. Relative FVLR values (%) are plotted against the threshold value (×VAD) for fovea (B) and parafovea (D) regions. P value of FVSC (E) and FVLR (F) between HC and DR for fovea and parafovea regions.

Table S1, P statistics for FVSC and FVLR between HC and DR for fovea and parafovea regions in DVP

Threshold	FVSC		FVLR	
(xVAD)	Fovea	Parafovea	Fovea	Parafovea
5	0.09250	0.00011	0.00051	0.00001
10	0.01327	0.00008	0.00014	0.00003
20	0.00134	0.00008	0.00006	0.00015
30	0.00069	0.00008	0.00007	0.00031
40	0.00106	0.00014	0.00004	0.00010
50	0.00073	0.00012	0.00004	0.00012
100	0.00011	0.00003	0.02752	0.00076
